# Supplementary material for: Prognostic Role of KRAS G12C Mutation in Non-Small Cell Lung Cancer: A Systematic Review and Meta-Analysis
Source: Diagnostics (Basel). 2023 Sep 25;13(19):3043. doi: 10.3390/diagnostics13193043 (PMC10572143; doi:10.3390/diagnostics13193043)
Supplement: Supplementary file 1 [file diagnostics-13-03043-s001.zip › diagnostics-2590728-supplementary.pdf]

Supplemental Online Content

Section and Topic	Item #	Checklist item	Location where item is reported
**TITLE**			
Title	1	Identify the report as a systematic review.	1
**ABSTRACT**			
Abstract	2	See the PRISMA 2020 for Abstracts checklist.	Abstract
**INTRODUCTION**			
Rationale	3	Describe the rationale for the review in the context of existing knowledge.	3
Objectives	4	Provide an explicit statement of the objective(s) or question(s) the review addresses.	3
**METHODS**			
Eligibility criteria	5	Specify the inclusion and exclusion criteria for the review and how studies were grouped for the syntheses.	4
Information sources	6	Specify all databases, registers, websites, organisations, reference lists and other sources searched or consulted to identify studies. Specify the date when each source was last searched or consulted.	4
Search strategy	7	Present the full search strategies for all databases, registers and websites, including any filters and limits used.	4
Selection process	8	Specify the methods used to decide whether a study met the inclusion criteria of the review, including how many reviewers screened each record and each report retrieved, whether they worked independently, and if applicable, details of automation tools used in the process.	4
Data collection process	9	Specify the methods used to collect data from reports, including how many reviewers collected data from each report, whether they worked independently, any processes for obtaining or confirming data from study investigators, and if applicable, details of automation tools used in the process.	5
Data items	10a	List and define all outcomes for which data were sought. Specify whether all results that were compatible with each outcome domain in each study were sought (e.g. for all measures, time points, analyses), and if not, the methods used to decide which results to collect.	5
	10b	List and define all other variables for which data were sought (e.g. participant and intervention characteristics, funding sources). Describe any assumptions made about any missing or unclear information.	5,6
Study risk of bias assessment	11	Specify the methods used to assess risk of bias in the included studies, including details of the tool(s) used, how many reviewers assessed each study and whether they worked independently, and if applicable, details of automation tools used in the process.	6
Effect measures	12	Specify for each outcome the effect measure(s) (e.g. risk ratio, mean difference) used in the synthesis or presentation of results.	5,6
Synthesis methods	13a	Describe the processes used to decide which studies were eligible for each synthesis (e.g. tabulating the study intervention characteristics and comparing against the planned groups for each synthesis (item #5)).	6
	13b	Describe any methods required to prepare the data for presentation or synthesis, such as handling of missing summary statistics, or data conversions.	6
	13c	Describe any methods used to tabulate or visually display results of individual studies and syntheses.	6
	13d	Describe any methods used to synthesize results and provide a rationale for the choice(s). If meta-analysis was performed, describe the model(s), method(s) to identify the presence and extent of statistical heterogeneity, and software package(s) used.	6

Section and Topic	Item #	Checklist item	Location where item is reported
	13e	Describe any methods used to explore possible causes of heterogeneity among study results (e.g. subgroup analysis, meta-regression).	6
	13f	Describe any sensitivity analyses conducted to assess robustness of the synthesized results.	6
Reporting bias assessment	14	Describe any methods used to assess risk of bias due to missing results in a synthesis (arising from reporting biases).	5,6
Certainty assessment	15	Describe any methods used to assess certainty (or confidence) in the body of evidence for an outcome.	-
**RESULTS**			
Study selection	16a	Describe the results of the search and selection process, from the number of records identified in the search to the number of studies included in the review, ideally using a flow diagram.	6
	16b	Cite studies that might appear to meet the inclusion criteria, but which were excluded, and explain why they were excluded.	Supplemental file
Study characteristics	17	Cite each included study and present its characteristics.	6
Risk of bias in studies	18	Present assessments of risk of bias for each included study.	6
Results of individual studies	19	For all outcomes, present, for each study: (a) summary statistics for each group (where appropriate) and (b) an effect estimate and its precision (e.g. confidence/credible interval), ideally using structured tables or plots.	7
Results of syntheses	20a	For each synthesis, briefly summarise the characteristics and risk of bias among contributing studies.	7
	20b	Present results of all statistical syntheses conducted. If meta-analysis was done, present for each the summary estimate and its precision (e.g. confidence/credible interval) and measures of statistical heterogeneity. If comparing groups, describe the direction of the effect.	7
	20c	Present results of all investigations of possible causes of heterogeneity among study results.	7,8
	20d	Present results of all sensitivity analyses conducted to assess the robustness of the synthesized results.	8
Reporting biases	21	Present assessments of risk of bias due to missing results (arising from reporting biases) for each synthesis assessed.	Not reported
Certainty of evidence	22	Present assessments of certainty (or confidence) in the body of evidence for each outcome assessed.	Not reported
**DISCUSSION**			
Discussion	23a	Provide a general interpretation of the results in the context of other evidence.	8
	23b	Discuss any limitations of the evidence included in the review.	8,9
	23c	Discuss any limitations of the review processes used.	9,10
	23d	Discuss implications of the results for practice, policy, and future research.	9,10
**OTHER INFORMATION**			

Section and Topic	Item #	Checklist item	Location where item is reported
Registration and protocol	24a	Provide registration information for the review, including register name and registration number, or state that the review was not registered.	OSF
	24b	Indicate where the review protocol can be accessed, or state that a protocol was not prepared.	OSF
	24c	Describe and explain any amendments to information provided at registration or in the protocol.	None
Support	25	Describe sources of financial or non-financial support for the review, and the role of the funders or sponsors in the review.	No funding source
Competing interests	26	Declare any competing interests of review authors.	No competing interest
Availability of data, code and other materials	27	Report which of the following are publicly available and where they can be found: template data collection forms; data extracted from included studies; data used for all analyses; analytic code; any other materials used in the review.	Data extracted from included studies

Table 1 PRISMA Checklist

Study ID	Number of stars			
	Selection /4	Comparability /2	Outcome or Exposure* /3	Total score /9
Arbour, 2021	4	2	2	8
Aredo, 2019	4	2	3	9
Cai, 2019	3	1	2	6
Cui, 2020	2	1	2	5
Finn, 2021	4	2	3	9
Jones, 2021	3	2	3	8
Liu, 2020	3	2	1	6
Nadal, 2014	4	2	3	9
Osta, 2019	4	2	3	9
Sebastian, 2021	2	2	2	6
Sun, 2013	2	2	2	6
Svanton, 2016	2	1	2	5
Tao, 2020	2	2	2	6
Villacruz, 2013	3	2	1	6
Wahl, 2021	4	2	3	9
Yu, 2015	3	1	2	6

Table 2. Newcastle-Ottawa Scale for study quality assessment

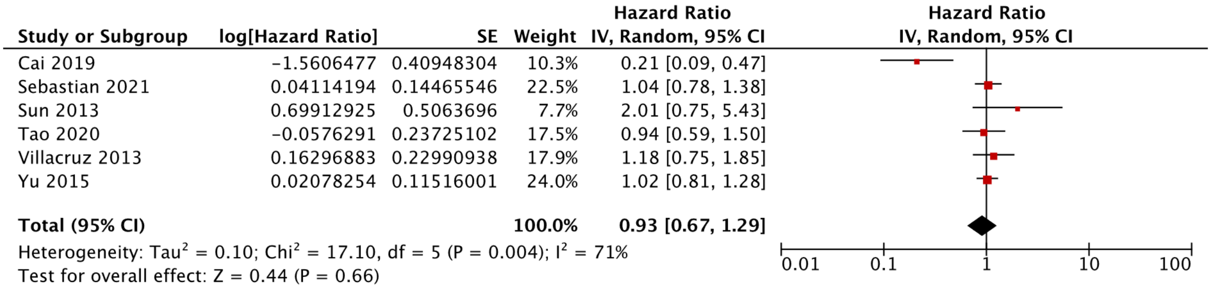

Figure 1. Additional analysis: Overall Survival, *KRAS G12C* vs *KRAS G12D* mutated tumors

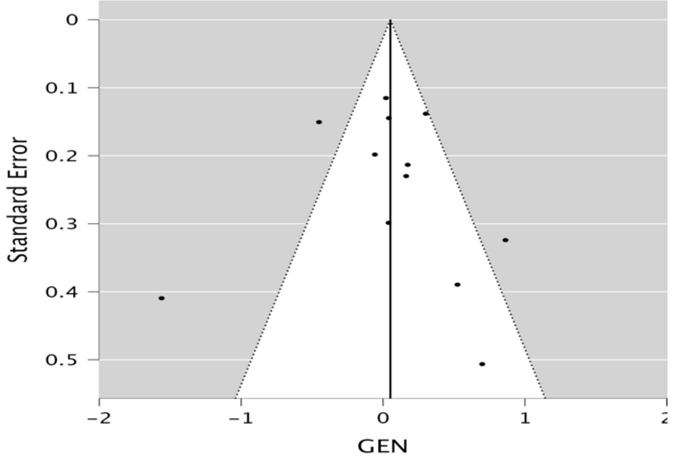

Figure 2. Funnel plot: Overall Survival *KRAS G12C* vs other *KRAS* mutations

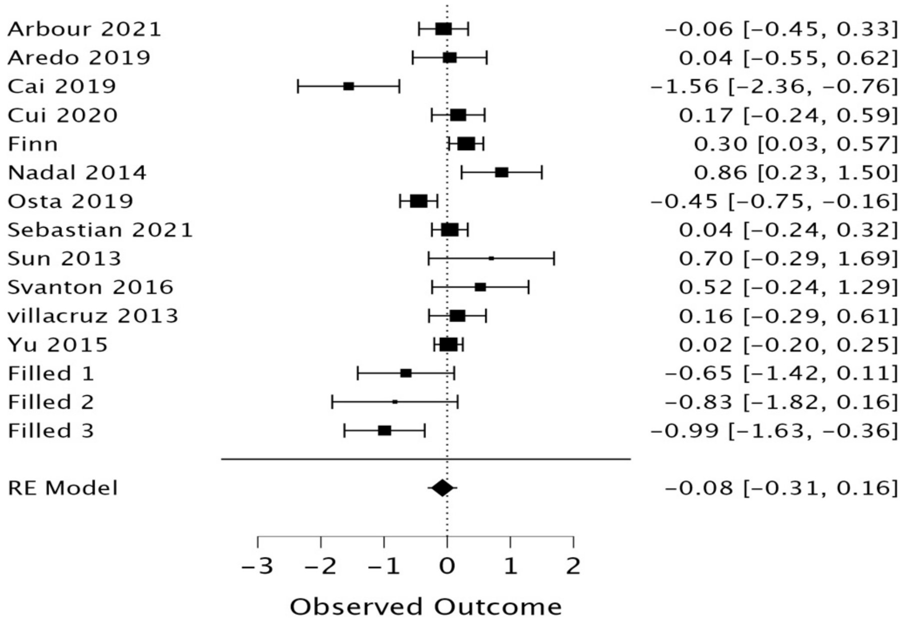

Figure 3 Trim-and-fill analysis Overall Survival KRAS G12C vs other *KRAS* mutations

Study or Subgroup	log[Hazard Ratio]	SE	Weight	Hazard Ratio IV, Random, 99% CI
**4.1.1 Asian**				
Cai 2019	−1.5606477	0.40948304	4.2%	0.21 [0.07, 0.60]
Sun 2013	0.69912925	0.5063696	3.1%	2.01 [0.55, 7.41]
**Subtotal (95% CI)**			**7.3%**	**0.64 [0.07, 5.83]**
Heterogeneity: Tau² = 2.34; Chi² = 12.04, df = 1 (P = 0.0005); I² = 92%				
Test for overall effect: Z = 0.40 (P = 0.69)				
**4.1.2 Non-asian**				
Arbour 2021	−0.0576291	0.1982216	8.3%	0.94 [0.57, 1.57]
Aredo 2019	0.03922071	0.29863919	6.0%	1.04 [0.48, 2.24]
Cui 2020	0.17395331	0.21332858	7.9%	1.19 [0.69, 2.06]
Finn 2021	0.30110509	0.13824393	9.9%	1.35 [0.95, 1.93]
Nadal 2014	0.86288996	0.32406201	5.5%	2.37 [1.03, 5.46]
Osta 2019	−0.4509856	0.15054161	9.5%	0.64 [0.43, 0.94]
Sebastian 2021	0.04114194	0.14465546	9.7%	1.04 [0.72, 1.51]
Svanton 2016	0.52472853	0.38952518	4.4%	1.69 [0.62, 4.61]
Tao 2020	−0.1851255	0.2144307	7.9%	0.83 [0.48, 1.44]
Villacruz 2013	0.16296883	0.22990938	7.5%	1.18 [0.65, 2.13]
Wahl 2021	−0.0304592	0.3228741	5.5%	0.97 [0.42, 2.23]
Yu 2015	0.02078254	0.11516001	10.4%	1.02 [0.76, 1.37]
**Subtotal (95% CI)**			**92.7%**	**1.06 [0.90, 1.26]**
Heterogeneity: Tau² = 0.04; Chi² = 24.30, df = 11 (P = 0.01); I² = 55%				
Test for overall effect: Z = 0.73 (P = 0.47)				
**Total (95% CI)**			**100.0%**	**1.03 [0.84, 1.26]**
Heterogeneity: Tau² = 0.09; Chi² = 41.09, df = 13 (P < 0.0001); I² = 68%				
Test for overall effect: Z = 0.27 (P = 0.79)				
Test for subgroup differences: Chi² = 0.21, df = 1 (P = 0.65), I² = 0%				

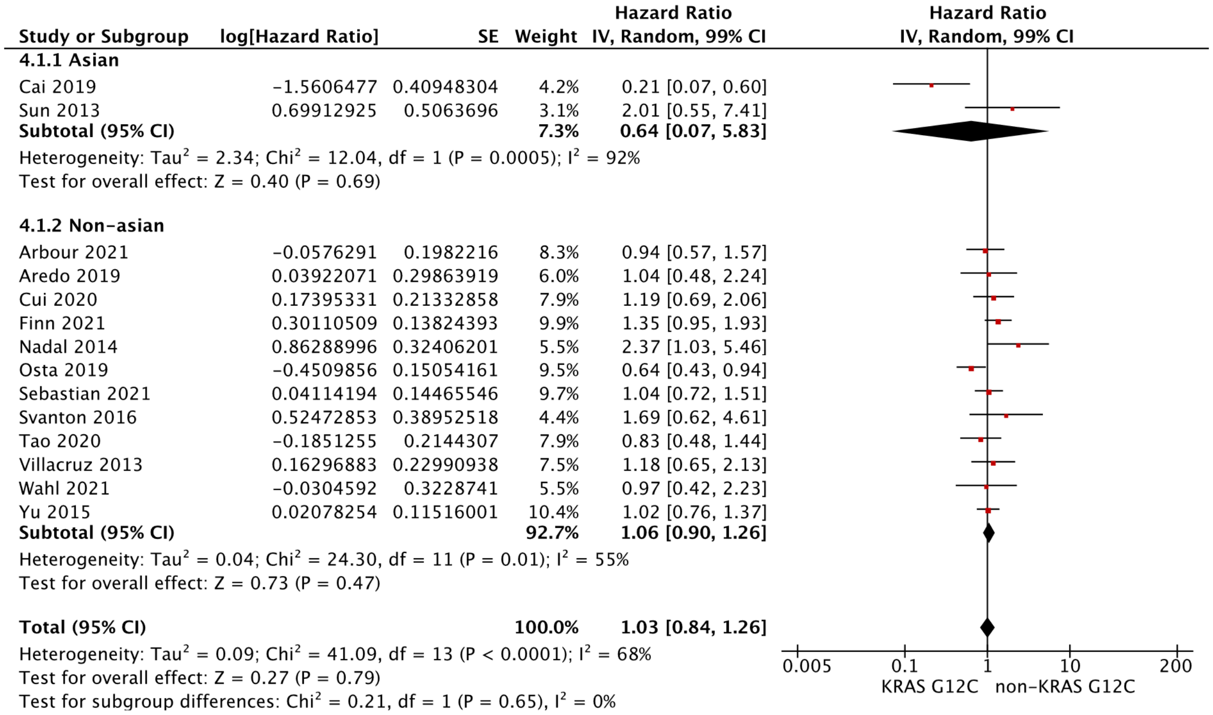

Figure 4. Subgroup analysis: ethnicity, Overall Survival KRAS G12C vs other *KRAS* mutations

Study or Subgroup	log[Hazard Ratio]	SE	Weight	Hazard Ratio IV, Random, 99% CI
**4.4.1 NGS**				
Arbour 2021	−0.0576291	0.1982216	9.1%	0.94 [0.57, 1.57]
Aredo 2019	0.03922071	0.29863919	7.2%	1.04 [0.48, 2.24]
Cui 2020	−1.5606477	0.40948304	5.5%	0.21 [0.07, 0.60]
Osta 2019	−0.4509856	0.15054161	9.9%	0.64 [0.43, 0.94]
Sebastian 2021	0.04114194	0.14465546	10.0%	1.04 [0.72, 1.51]
Wahl 2021	−0.0304592	0.3228741	6.8%	0.97 [0.42, 2.23]
**Subtotal (95% CI)**			**48.6%**	**0.77 [0.55, 1.08]**
Heterogeneity: Tau² = 0.12; Chi² = 18.03, df = 5 (P = 0.003); I² = 72%				
Test for overall effect: Z = 1.50 (P = 0.13)				
**4.4.2 PCR-sequence**				
Cai 2019	−1.5606477	0.40948304	5.5%	0.21 [0.07, 0.60]
Finn 2021	0.30110509	0.13824393	10.1%	1.35 [0.95, 1.93]
Nadal 2014	0.86288996	0.32406201	6.8%	2.37 [1.03, 5.46]
Sun 2013	0.69912925	0.5063696	4.3%	2.01 [0.55, 7.41]
Svanton 2016	0.52472853	0.38952518	5.8%	1.69 [0.62, 4.61]
Villacruz 2013	0.16296883	0.22990938	8.5%	1.18 [0.65, 2.13]
Yu 2015	0.02078254	0.11516001	10.5%	1.02 [0.76, 1.37]
**Subtotal (95% CI)**			**51.4%**	**1.17 [0.80, 1.70]**
Heterogeneity: Tau² = 0.17; Chi² = 26.85, df = 6 (P = 0.0002); I² = 78%				
Test for overall effect: Z = 0.80 (P = 0.42)				
**Total (95% CI)**			**100.0%**	**0.95 [0.73, 1.23]**
Heterogeneity: Tau² = 0.16; Chi² = 54.28, df = 12 (P < 0.00001); I² = 78%				
Test for overall effect: Z = 0.38 (P = 0.70)				
Test for subgroup differences: Chi² = 2.55, df = 1 (P = 0.11), I² = 60.9%				

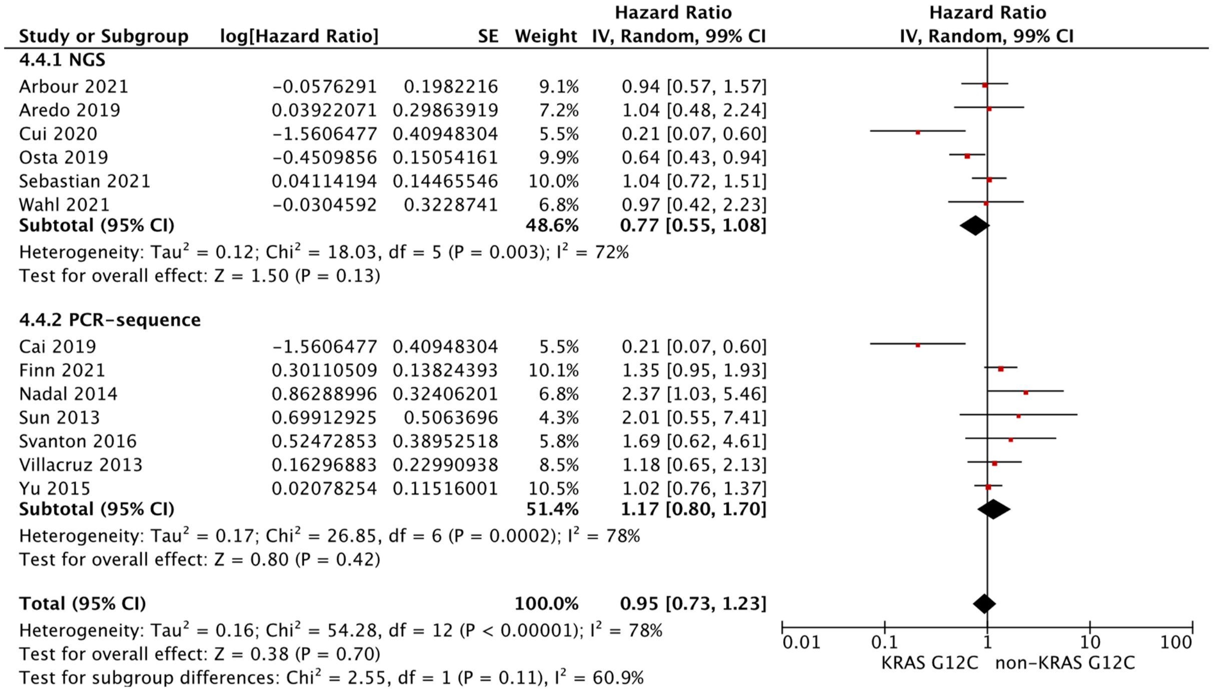

Figure 5. Subgroup analysis: Testing methods, Overall Survival KRAS G12C vs other *KRAS* mutations

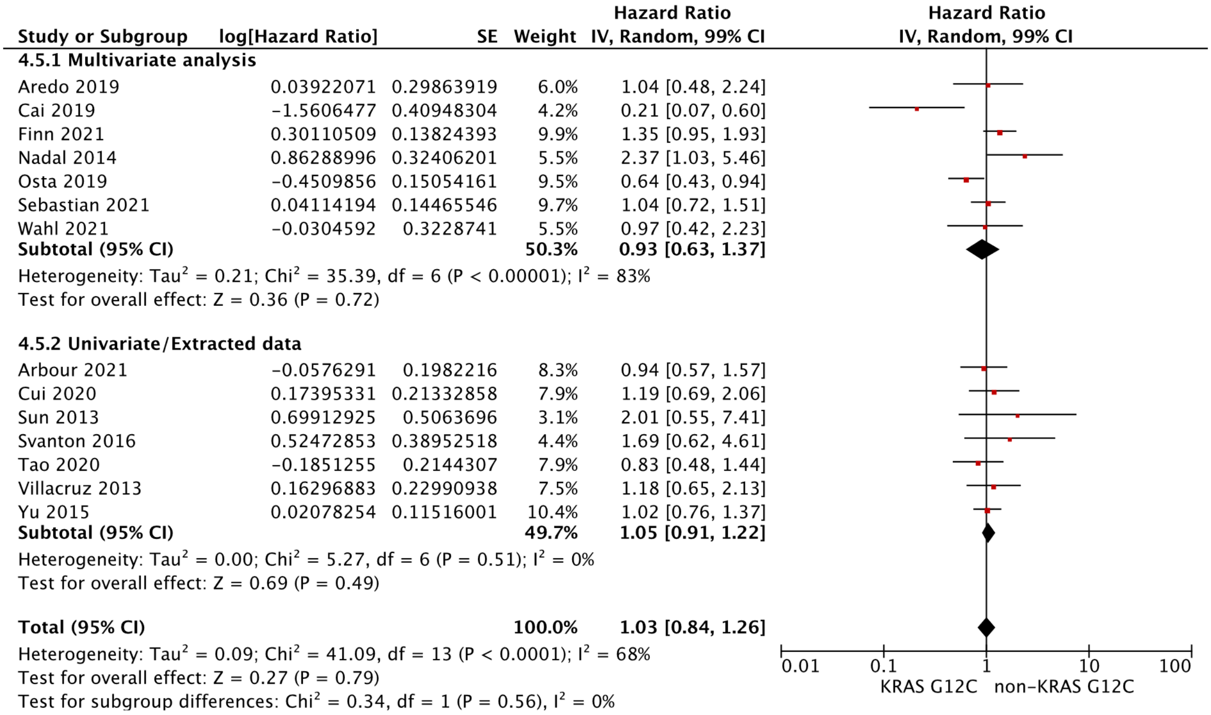

Figure 6. Subgroup analysis: data source: Overall Survival KRAS G12C vs other *KRAS* mutations

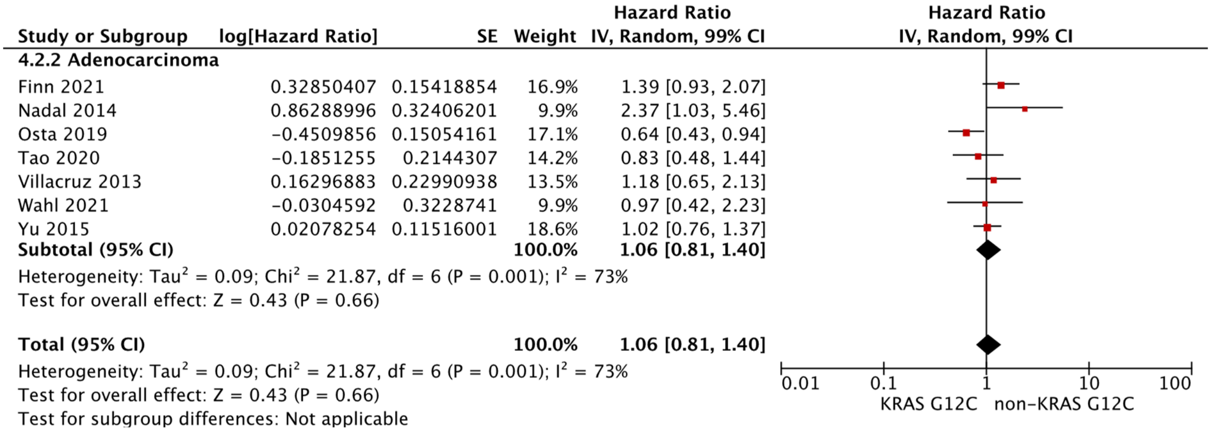

Figure 7. Additional analysis: Histology: Overall Survival KRAS G12C vs other *KRAS* mutations

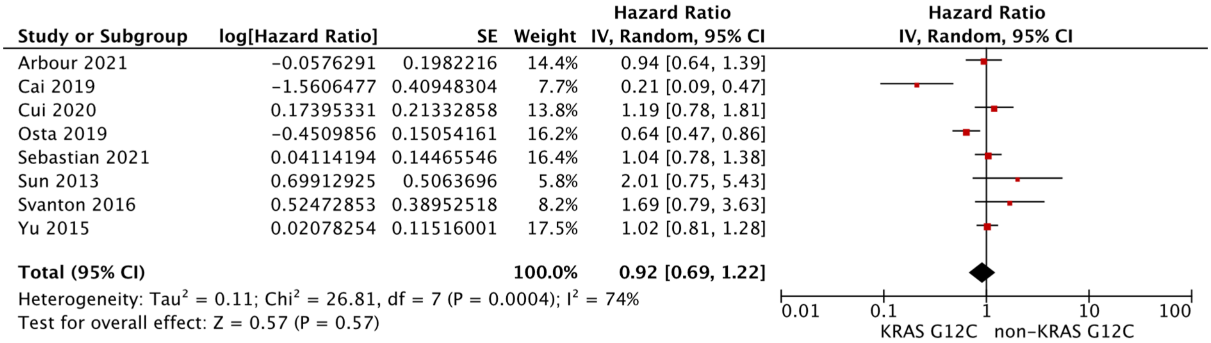

Figure 8. Additional analysis: Advanced Stage, Overall Survival KRAS G12C vs other *KRAS* mutations

Study or Subgroup	log[Hazard Ratio]	SE	Weight	Hazard Ratio IV, Random, 95% CI
Tao 2020	−0.0855579	0.1606106	54.6%	0.92 [0.67, 1.26]
Villacruz 2013	0.7756484	0.30614956	45.4%	2.17 [1.19, 3.96]
**Total (95% CI)**			**100.0%**	**1.36 [0.59, 3.15]**

Heterogeneity: Tau² = 0.31; Chi² = 6.21, df = 1 (P = 0.01); I² = 84%
Test for overall effect: Z = 0.71 (P = 0.48)

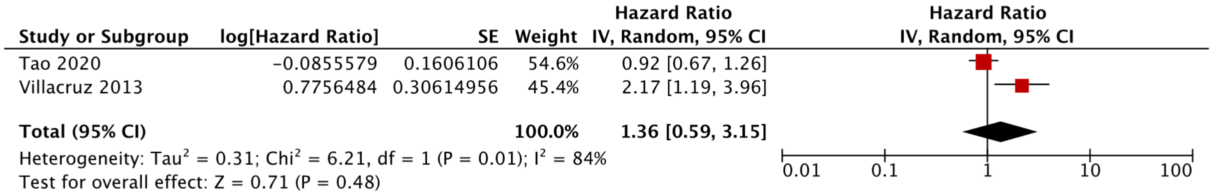

Figure 9 Additional analysis: Disease-free Survival, *KRAS G12C* vs *KRAS G12D* mutated tumors

Study or Subgroup	KRAS G12C Events	KRAS G12C Total	Other KRAS mutations Events	Other KRAS mutations Total	Weight	Odds Ratio M-H, Fixed, 95% CI
Arbour 2021	167	352	212	418	47.1%	0.88 [0.66, 1.16]
Cui 2020	2	65	9	79	3.6%	0.25 [0.05, 1.19]
Finn 2021	84	216	102	257	26.3%	0.97 [0.67, 1.40]
Jones 2021	45	95	65	135	13.1%	0.97 [0.57, 1.64]
Sebastian 2021	10	160	29	251	9.8%	0.51 [0.24, 1.08]
**Total (95% CI)**		**888**		**1140**	**100.0%**	**0.85 [0.70, 1.04]**
Total events	308		417			

Heterogeneity: Chi² = 4.91, df = 4 (P = 0.30); I² = 19%
Test for overall effect: Z = 1.58 (P = 0.12)

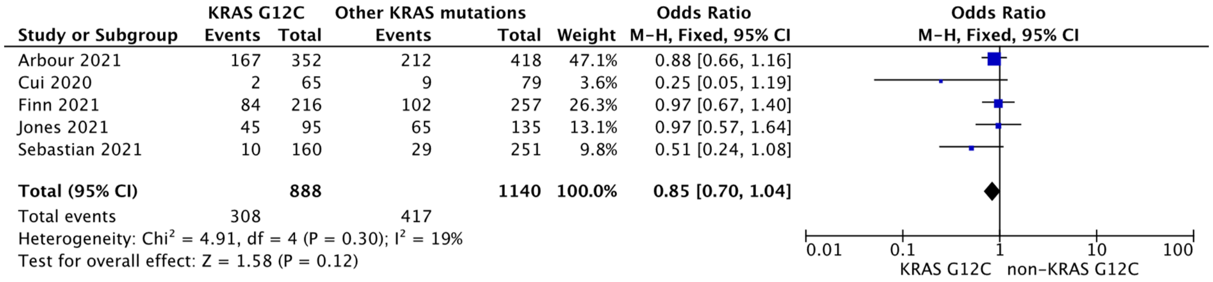

Figure 10. PD-L1 expression <1%

Study or Subgroup	KRAS G12C Events	KRAS G12C Total	Other KRAS mutations Events	Other KRAS mutations Total	Weight	Odds Ratio M-H, Fixed, 95% CI
Arbour 2021	49	352	67	418	33.6%	0.85 [0.57, 1.26]
Cui 2020	9	65	9	79	4.5%	1.25 [0.47, 3.36]
Finn 2021	46	216	61	257	28.0%	0.87 [0.56, 1.34]
Jones 2021	15	95	23	135	10.2%	0.91 [0.45, 1.86]
Sebastian 2021	46	160	67	251	23.7%	1.11 [0.71, 1.72]
**Total (95% CI)**		**888**		**1140**	**100.0%**	**0.94 [0.75, 1.18]**
Total events	165		227			

Heterogeneity: Chi² = 1.24, df = 4 (P = 0.87); I² = 0%
Test for overall effect: Z = 0.54 (P = 0.59)

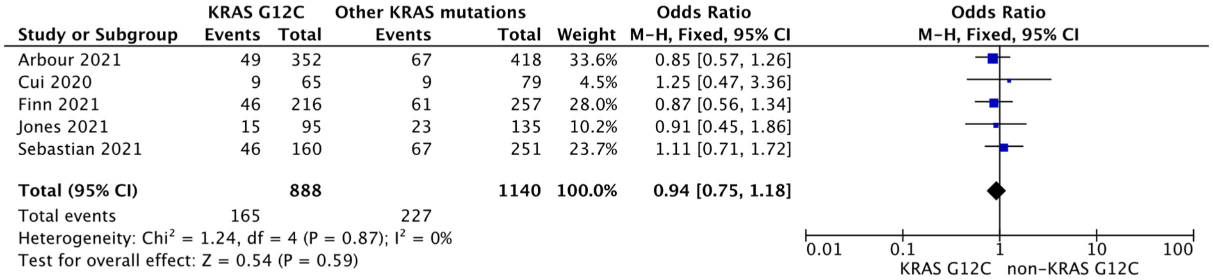

Figure 11. PD-L1 expression 1-49%

Study or Subgroup	KRAS G12C Events	KRAS G12C Total	Other KRAS mutations Events	Other KRAS mutations Total	Weight	Odds Ratio M-H, Random, 95% CI
Arbour 2021	151	352	171	418	39.7%	1.09 [0.81, 1.45]
Jones 2021	24	95	40	135	27.3%	0.80 [0.44, 1.45]
Lei 2020	11	25	12	50	15.1%	2.49 [0.90, 6.91]
Nadal 2014	17	35	34	50	18.0%	0.44 [0.18, 1.08]
**Total (95% CI)**		**507**		**653**	**100.0%**	**0.96 [0.59, 1.56]**
Total events	203		257			

Heterogeneity: Tau² = 0.13; Chi² = 7.07, df = 3 (P = 0.07); I² = 58%
Test for overall effect: Z = 0.15 (P = 0.88)

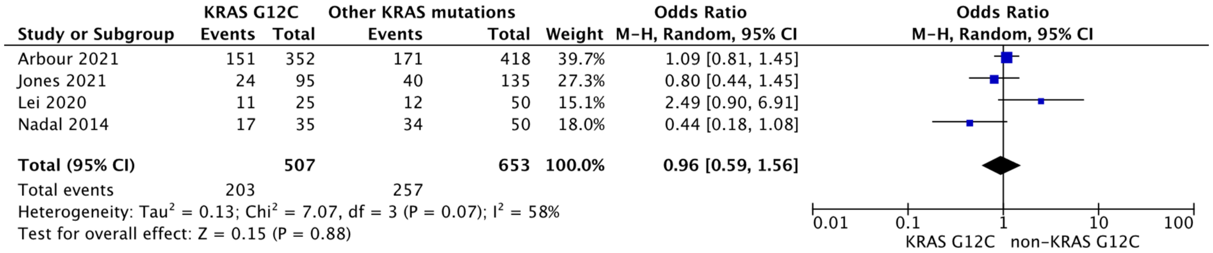

Figure 12. TP53 mutation

Study or Subgroup	KRAS G12C Events	KRAS G12C Total	Other KRAS mutations Events	Other KRAS mutations Total	Weight	Odds Ratio M-H, Fixed, 95% CI
Arbour 2021	99	352	121	418	75.9%	0.96 [0.70, 1.31]
Jones 2021	21	95	20	135	12.3%	1.63 [0.83, 3.22]
Lei 2020	0	25	4	50	2.8%	0.20 [0.01, 3.92]
Nadal 2014	10	35	16	50	9.0%	0.85 [0.33, 2.18]
**Total (95% CI)**		**507**		**653**	**100.0%**	**1.01 [0.77, 1.32]**
Total events	130		161			

Heterogeneity: Chi² = 3.28, df = 3 (P = 0.35); I² = 8%
Test for overall effect: Z = 0.08 (P = 0.93)

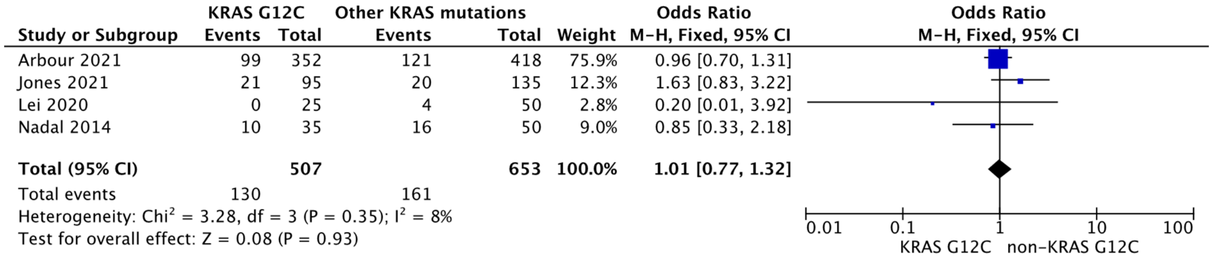

Figure 13. STK11 mutation.
